# Risk of Fungi Associated with Aflatoxin and Fumonisin in Medicinal Herbal Products in the Kenyan Market

**DOI:** 10.1155/2017/1892972

**Published:** 2017-08-08

**Authors:** Lucia Keter, Richard Too, Nicholas Mwikwabe, Charles Mutai, Jennifer Orwa, Lizzy Mwamburi, Stanley Ndwigah, Christine Bii, Richard Korir

**Affiliations:** ^1^Kenya Medical Research Institute, P.O. Box 54840-00200, Nairobi, Kenya; ^2^Department of Biological Sciences, University of Eldoret, P.O. Box 1125-30100, Eldoret, Kenya; ^3^Masinde Muliro University of Science and Technology, P.O. Box 190-50100, Kakamega, Kenya; ^4^Department of Pharmaceutical Chemistry, School of Pharmacy, University of Nairobi, P.O. Box 19676-00202, Nairobi, Kenya

## Abstract

Utilization of herbal products is a major concern due to the possibility of contamination by toxigenic fungi that are mycotoxin producers such as* Aspergillus* species during processing and packaging. Research was carried out to determine the presence of aflatoxins and fumonisins in herbal medicinal products sold in Eldoret and Mombasa towns in Kenya. The study employed both exploratory and laboratory experimental design. The herbal products were purchased from the market and transported to Kenya Medical Research Institute for processing and analysis. Fungal contaminants were determined according to Pharmacopoeia specifications. The toxins were quantified using ELISA based technique. The genus* Aspergillus* was the most dominant followed by* Penicillium*. Fungal counts ranged between 1 CFU/g and >1000 cfu/g. Analysis of variance showed that the rate of fungal contaminants for Eldoret and Mombasa samples had significant association (*p* ≤ 0.001). Aflatoxin levels ranged from 1 to 24 ppb, while fumonisin levels ranged from 1 to >20 ppb. Only 31% of samples met the standards for microbial limits as specified in Pharmacopoeia. There is need for product microbial quality improvement through proper harvesting, processing, storage, and marketing. It is recommended that a policy be enacted to enable regulation of herbal products in Kenya.

## 1. Introduction

In recent years, there has been a general increase in consumption of herbal products and over the counter (OTC) health foods, nutraceuticals, and medicinal products from plants or other natural sources in developed countries [1]. This increase in the consumption of these herbal products has become a public health issue. Practices of cultivation, harvest, storage, processing, handling, and distribution together with the absence of effective sanitary control measures make natural products subject to a great variety of contamination. Of concern there also is the professionalism of practitioners in terms of quality, efficacy, and safety of their treatment methods and products from herbal and natural sources available in the market [2]. The interest in safety of these products is greatly due to the possible presence of pathogenic bacteria and toxigenic fungi that produce mycotoxins such as aflatoxins, excessive or banned pesticides, microbial contaminants, heavy metals, chemical toxins, or adulteration with conventional drugs [3]. The toxic effects of the aflatoxins include immunosuppressive, mutagenic, teratogenic, and hepatocarcinogenic activity.

A World Health Organization (WHO) survey indicated that about 70–80% of the world populations rely on nonconventional medicine mainly of herbal sources in their primary healthcare [4]. Most consumers and vendors believe and consider herbal products to be safe but microbial contamination in medicinal herbs is a concern, especially among the immunocompromised individuals as a result of their lowered immunity [5]. Medicinal herbal products have been reported to be contaminated with microorganisms indigenous to the soil and plants where they are grown [6]. Poor conditions during harvesting and postharvest handling of the herbs and herbal products predispose them to contamination [7]. Fungal contaminations of herbal products chiefly occur during a slow drying process as a result of inadequate drying or during postharvest storage if relative humidity is high or if temperatures are favourable. The fungal contaminants are primarily of environmental origin especially from fungal spores found in the soil and air. Such contaminated products may cause fungal infections or other serious health complications as a result of mycotoxins accumulation from toxin-producing fungi such as* Aspergillus parasiticus* and* Aspergillus flavus* [8]. Furthermore, several studies have reported the presence of mycotoxins in botanical preparations [7, 9] and elevated levels of fungal contaminants, such as* Penicillium* sp.,* Aspergillus* sp., and* Fusarium* sp., have been observed in herbal drugs and spices [7]. Due to the absence of data about the occurrence of mycotoxins in natural products in Kenya, this paper aimed at evaluating the occurrence of aflatoxins and fumonisins contaminants in some herbal products marketed in Eldoret and Mombasa towns.

## 2. Materials and Methods

### 2.1. Sample Collection

Herbal products were sampled from hawkers/street vendors, herbal clinics, and manufacturers in Eldoret and Mombasa towns of Kenya. These towns were chosen because of the difference in weather conditions due to their geographical locations. Mombasa town is located in the coastal strip of Kenya, while Eldoret town is in the highlands of Rift valley. The former being hot and humid while the latter being cold and wet. The sites also enjoy different cultural diversity and the use of herbal products is different among various communities in these regions. The samples were purchased in their original pack with the exception of a few in which the liquid herbal products were collected and packaged in sterile containers while the powdered products were packed in sanitary brown envelopes and transparent polyethylene bags and transportation to the Kenya Medical Research Institute (KEMRI) laboratories for processing and analysis. The samples were assigned unique codes that were used in laboratory assays, data analysis, report writing, and presentation of the results for confidentiality [10]. The fungi and mycotoxins contaminations assays were carried out at KEMRI Centre for Microbiology Research laboratories. A total of 100 herbal products were sampled from the two study sites.

### 2.2. Determination of Fungal Contamination

Microbial contamination was determined according to Pharmacopoeia and WHO requirements [4, 11, 12]. Microbiological parameters considered in this study were total mould/yeast count and their count (colony forming units). One gram of each herbal product in powder, tablet, and capsule form was aseptically suspended in 1 ml of either hot or cold sterile distilled water (as per the label claim preparation instructions), mixed thoroughly, then topped to make 10 percent concentration, and sieved before serial dilutions were done. Appropriate serial dilutions were made and 0.1 ml of the dilution transferred aseptically to sterile Petri plates containing culture media, Sabouraud's Dextrose Agar (Oxoid, UK) supplemented with chloramphenicol to inhibit bacterial contamination. Double plates of each sample were incubated at 25 ± 2°C for 3–7 days and examined daily for fungal growth. After incubation, fungal counts were determined, and distinct colonies were subcultured. For the liquid and oils formulations, the initial concentrations on the label were noted and then serial dilution was carried out. The samples were transferred into the culture media and incubated as outlined above. Fungal colonies were counted and recorded accordingly.

### 2.3. Identification of the Isolated Fungi

To determine the kinds of fungi present, distinct fungal colonies were subcultured onto Sabouraud's Dextrose Agar (SDA) to obtain pure cultures for the purpose of identification. They were incubated at 25 ± 2°C until the colonies of fungi could be identified. Two kinds of media, potato dextrose agar (PDA) (Oxoid, UK) plates containing 100 *µ*g of chloramphenicol per ml and 20% glucose-PDA plates for isolation of xerophiic fungi, were used in this experiment. After growth of the cultures, identification was done using cultural, morphological, and microscopic charateristics. All isolates were maintained on PDA slants. Lactophenol cotton blue was used for staining fungal cell during microscopy. A dichotomouse key was used in identification of fungi to species level.

### 2.4. Determination of Aflatoxins and Fumonisins

Aflatoxins and fumonisins were determined from the 100 samples using QuickToxTM Kit (Envirologix Inc., USA) for QuickScan (aflatoxins and fumonisins, resp.) following the manufacturer's instructions. The main principle of these kits is antibody antigen reaction. This is a rapid test where the strips are incubated for 5 minutes and then put in an electronic reader which reads and presents the results in parts per billion in a computer monitor. Briefly, the toxins were extracted from the sample with 50% ethanol and shaken for 2 minutes and left to stand for five minutes. Approximately 100 *µ*l of the top layer was drawn and put in a small vial and then mixed with 100 *µ*l DB2 Buffer provided with the kit and mixed well before applying the strip and then incubated for 5 minutes. The two lines in the rapid strip indicated a positive test while one line indicated a negative test. The strips were inserted in a Quick Scan Machine reader and the concentrations of the fumonisins and aflatoxins were determined in parts per billion (ppb) [13]. The kit is used for screening of total aflatoxins and fumonisins.

### 2.5. Statistical Analysis

Data was entered into statistical package for social scientist (SPSS) version 19.0 for analysis. The microbial contamination data was analysed statistically using ANOVA and results compared with the Pharmacopoeia and WHO requirements. The mycotoxins concentration in ppb was analysed using ANOVA and Student's *t*-test. The *p* value ≤ 0.05 was considered significant.

## 3. Results

### 3.1. Level of Fungal Contamination

Five samples had some fungus growing after 12 hours of incubation. After three days (72 hours) of incubation, there were 22% (11/50) samples from Eldoret and 34% (17/50) samples from Mombasa that were free from any fungal contamination.

**Figure 1:**
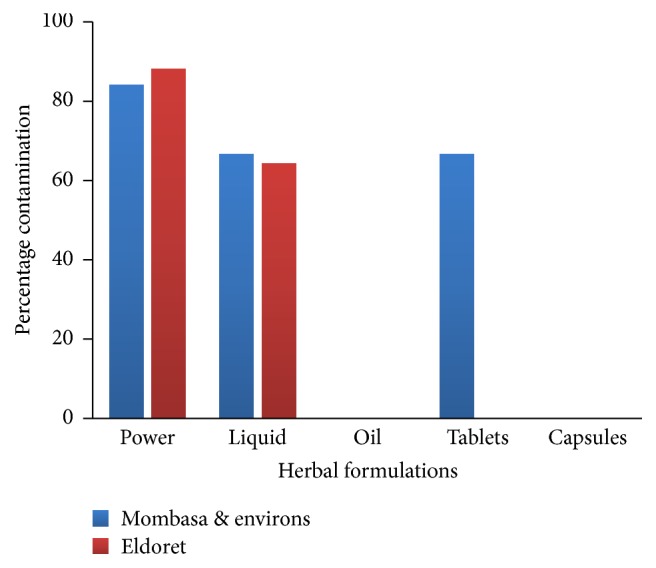
Percentage of the herbal products contaminated with fungi after 72 hours (*n*-100).

Thirty out of 34 (88.2%) of the powders and 9/14 (64.3%) of the liquids from Eldoret were contaminated while the oils and controls (conventional drugs, chlorphenamine, and paracetamol syrups) had no contamination. Mombasa herbal powders were more contaminated at 84.2% (16/19) followed by liquids at 66.7% (8/12), tablets 66.7% (10/15), while oils, capsules, and controls were not contaminated (Figure 1).

A mean fungi CFU after 72 hours was 1.46 × 10^5^ with a median of 5.0 × 10^4^ CFU/g or ml for all the samples. One sample from Eldoret had >1.0 × 10^9^ CFU/g or ml while, for Mombasa, 94% (47/50) samples had CFU of between 0–1.00 × 10^6^ CFU/g or ml and 2% had CFU of >9.00 × 10^6^ CFU/g or ml. For Eldoret samples, liquid formulation had a mean fungi CFU after 72 hour of 1.2 × 10^5^, oil formulation had 0, and powder formulation had 1.6 × 10^5^, while, for the Mombasa samples, the capsule and oil formulation had 0, liquid formulation had an average of 2.3 × 10^5^, powder formulation had 1.3 × 10^5^, and tablet formulation had 1.1 × 10^5^ CFU/g or ml (Table 1).

### 3.2. Identification of Fungal Isolates from the Herbal Products

The fungi were identified as environmental moulds and yeast. These organisms have been associated with those causing diseases in animals including humans. Some are found in the environment, while others are utilized in the industries especially* Saccharomyces* species. They were then classified and grouped into 14 genera:* Aspergillus*,* Penicillium*,* Saccharomyces*,* Rhizopus*,* Rhodotorula*,* Cryptococcus*,* Basidiobolus*,* Mucor*,* Malbranchea*,* Absidia*,* Trichophyton*,* Scedosporium*,* Fusarium*, and* Candida*.

**Figure 2:**
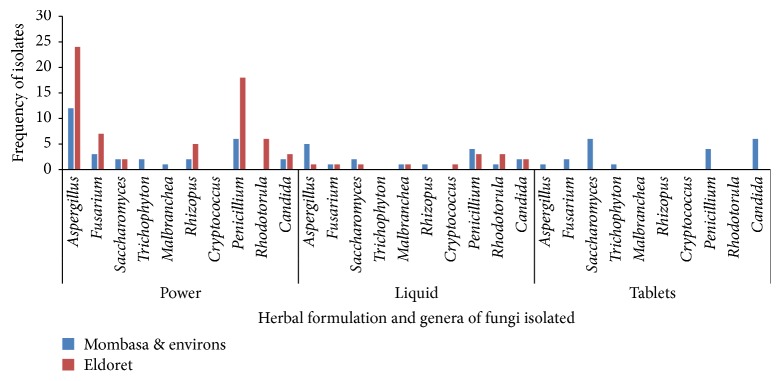
Fungi isolated from the different formulations of herbal products from Eldoret and Mombasa.

From Eldoret samples,* Penicillium* and* Rhodotorulla* were the most prevalent microorganisms in the liquids samples, while* Aspergillus* and* Penicillium* are the most frequent contaminants in the powdered samples. There was no contamination in the oils and capsules samples. From Mombasa samples,* Aspergillus* species were predominant in the liquid samples, followed by* Saccharomyces* sp. and* Candida* sp., while* Saccharomyces* and* Candida* dominated the isolates from tablets.* Aspergillus* and* Penicillium* were predominated in the powders. There were no contaminations in the oils and capsules (Figure 2).

### 3.3. Aflatoxin and Fumonisin Contamination from the Herbal Products

Thirty-two percent of Eldoret samples had aflatoxin levels <0.25 ppb, while 34% had aflatoxin level ranging from 0.38 to 24 ppb. Fumonisin levels were very low in more than half of the samples. For the Mombasa samples, aflatoxin contamination levels were lower than those of Eldoret samples, but the number of aflatoxin contaminated samples was higher. About 32% of the samples had <0.25 ppb with 14 ppb being the highest. About 80% had <0.25 ppb and the highest was >20 ppb.

Six out of 14 (42.9%) liquid samples from Eldoret were contaminated with aflatoxin and 3 of the 6 were also contaminated with fumonisin. All the 12 (100%) liquid samples from Mombasa were contaminated with both aflatoxins and fumonisins. A total of 27 out of 34 (79.4%) powders from Eldoret were contaminated, 23 with both mycotoxins and 4 with aflatoxin only, while all the tablets (15 samples) and powders (19 samples) from Mombasa were contaminated with both mycotoxins; however, the capsules were free of contamination. All the 3 oils samples from Mombasa were contaminated with both aflatoxin and fumonisin, while one oil sample from Eldoret was contaminated with fumonisin only.

Data analysis using *t*-test showed that the two mycotoxins tested from the two sites had varying significant levels. According to this test, aflatoxin from the two sites and fumonisin from Mombasa had significant difference, while fumonisin from Eldoret had no significant difference at 95% confidence interval which was the level of significance (*p* ≤ 0.05) (Table 2).

The variances were analysed between groups and within groups for both fumonisin and aflatoxin levels in the Eldoret and Mombasa samples using ANOVA. There was no significance (*p* > 0.05, *F* test > 0.05) association with the entire variable tested.

## 4. Discussion

The predominant mycoflora obtained in this study was distributed in 14 genera. The genus* Aspergillus* was the most dominant followed by* Penicillium*. These observations are in agreement with reports by other researchers [14–17] who investigated fungal contaminations of spices and herbal products. Anyanwu in a study reported* Aspergillus* spp. predominately occurring in 58.3% followed by* Penicillium* spp. occurring in 41.6% of the herbal samples [14]. The presence of a wide range of storage fungi indicates that considerable improvements could be made during postharvest storage. Strains of* Aspergillus flavus*,* A. niger*, and* Penicillium marneffei* were the most dominant and frequently isolated. These results closely compare with previous reports that showed* Aspergillus* species was the main contaminant in different herbal products and spices samples [15]. Most of the identified moulds have been reported to have ability to produce mycotoxins that are of grave consequence if consumed by both man and animals [18]. Korir and Bii [19] isolated the similar fungi in their study on mycological quality of maize floor from Makueni, Kitui, and Machakos Counties in Kenya formally Eastern province during the 2005 outbreak of aflatoxin in the region.

*Penicillium* and* Rhodotorulla* were predominant in the liquids from Eldoret herbal products samples, while* Aspergillus* and* Penicillium* were very common in the powders. In the Mombasa herbal products,* Aspergillus* species were predominat isolates from the liquid samples, followed by* Saccharomycess* species;* Candida* species and* Saccharomyces* dominated the tablets.* Aspergillus* and* Penicillium* were isolated in bulk from the powdered samples. Of great concern was the isolation of* Cryptococcus neoformans* from the herbal products. This pathogen causes cryptococcal meningitis especially in immunocompromised individuals and can be of grave consequences bearing in mind that the herbalists claimed to treat a wide array of ailments including HIV/AIDS using the products. Although high fungal loads may be accepted due to the natural origin of these products, they indicate the potential for spoilage and mycotoxin contamination [15]. Considering natural flora, current production conditions, and the need to warrant the quality and the safety of these products, monographs of the US Pharmacopoeia for products that contain raw material of natural origin establish a maximum fungal contamination limit of 10^5^/10^4^ CFU/ml of the product [12]. In the current study, fungal contamination from the two sites had colony forming units of between 1.0 × 10^0^ CFU/ml and <1.0 × 10^9^ CFU/ml with 69% being above the US Pharmacopoeia limits. It was noted that samples that contained* Aloe vera* concoctions were free from fungi contaminants. This is possibly because* Aloe vera* has been reported to have high antifungal properties [20].

The presence of moulds in a sample was not associated with the presence of mycotoxins. This is because fungus can produce toxins and die, therefore failing to grow in culture media, yet the toxins would still be in the product [21]. From this study, it is evident that herbal medicinal products sold in Kenya are highly contaminated with mycotoxins and microbes that are potential pathogens, hence posing a threat to the final consumers (both healthy and sick persons). Most of the identified moulds have been reported to have ability to produce mycotoxins in previous studies [19, 22]. Analysis of fumonisin and aflatoxin showed that, from Eldoret, 32% had <0.25 ppb, while 34% had between 0.38 ppb and 24 ppb of aflatoxin. When aflatoxin levels were high in a sample, fumonisin was very low. Mombasa samples fumonisin levels were also low as compared to Eldoret samples. Bugno et al. [18] in their study on fungal contamination of herbal products found that 49.0% of the isolates produced high levels of aflatoxins. The *t*-test showed that aflatoxins and fumonisins from the two sites had statistical significant association (*p* ≤ 0.05); hence there were no significant differences. The herbal preparations in this study revealed the presence of mycotoxins; thus there is a potential risk for contamination, especially during prolonged storage in poor conditions without temperature and moisture control that usually render medicinal plants more susceptible to moulds growth and mycotoxins production [18]. Such contaminated products may cause fungal infections or other serious health complications as a result of mycotoxin accumulation from toxin-producing fungi such as* Aspergillus parasiticus* and* A. flavus*. Similar studies have observed the presence of mycotoxins in botanical preparations [13, 23].

Herbalists interviewed in this study claimed to treat different ailments with the herbs and thus antifungal properties of the herbs could reduce fungal contaminants even in poorly handled and stored products. On the basis of the results, the microbiological quality of the herbal products is influenced to varying degrees by the microbial levels of the starting raw materials, probably the production method and the production environment. Some of these environment related factors can be controlled by implementing standard operating procedures (SOP) leading to Good Agricultural Practice (GAP), Good Manufacturing Practice (GMP), Good Laboratory Practice (GLP), and Good Supply Practice (GSP) for producing these medicinal products from herbal or natural sources. The general population believes that herbal and natural products are safer than synthetic medicines, yet the current study has found that some of the products are contaminated beyond the international accepted limits. Quality of the berbal products can only be ascertained by imposing regulatory standards on these products using the aforementioned good practices [24].

## 5. Conclusion

Most of the herbal products (69%) did not comply with the fungal acceptable limits as outlined in international Pharmacopoeias. Of great concern was the isolation of* Cryptococcus neoformans*, a highly pathogenic fungus that mainly affect the lungs and central nervous system of persons with compromised immunity. The presence of toxigenic fungi necessitates the need for standardization of herbal products given the global acceptance of these products as neutraceuticals and remedies for various diseases.

## Recommendations

Further research could be carried out to analyse other toxins such as ochratoxin, zearalenone, and deoxynivalenol (DON) among other potential mycotoxins associated with mycotoxigenic fungi isolated.

Considering the worldwide increased use of herbal products as alternative medicines and the risk of purchase and use of natural products contaminated with moulds and mycotoxins, it is necessary to put up regulatory and legislative mechanisms. It is therefore recommended that a policy be established to enable regulation of the quality of herbal medicinal products sold in the Kenya market.

## Figures and Tables

**Table 1:** Fungal colony forming units per gram (CFU/g) of Mombasa and Eldoret samples after 72 hours incubation (*n*-100).

Site	Formulation form	Mean (10^4^)	*N*	Std. deviation	Median (10^4^)	Sum (10^4^)	Min (10^4^)	Max (10^4^)	*p* value (*F*-value)
Eldoret	Liquid	11.86	14	21.775	1.00	166	0	52	0.965 (0.001)
	Oil	0.00	2	0.000	0.00	0	0	0	
	Powder	15.68	34	15.503	10.00	533	0	52	

Mombasa	Capsules	0.00	1		0.00	0	0	0	0.965 (0.001)
	Liquid	22.50	12	21.865	20.50	270	0	52	
	Oil	0.00	3	0.000	0.00	0	0	0	
	Powder	13.25	28	18.940	6.50	371	0	52	
	Tablets	11.00	6	19.637	2.00	66	0	50	

*Note*. Min: minimum; Max: maximum; *N*: total sample size per formulation; Std: standard deviation.

**Table 2:** Analysis of aflatoxin and fumonisin for Eldoret and Mombasa samples using Student's *t*-test (*n*-100).

One-sample test	*t*	Df	Significance (2-tailed)	Mean difference	95% confidence interval of the difference	
					Lower	Upper
Aflatoxin ELD	3.153	52	0.003	2.099	0.763	3.434
Fumonisin ELD	1.559	52	0.125	12.341	3.548	28.229
Aflatoxin MSA	5.040	52	0.001	1.718	1.034	2.402
Fumonisin MSA	3.535	52	0.001	22.132	9.568	34.696

ELD: Eldoret; MSA: Mombasa; df: degree of freedom; *t*: Student's *t*-test.
